# Line-scanning Brillouin microscopy for rapid non-invasive mechanical imaging

**DOI:** 10.1038/srep35398

**Published:** 2016-10-14

**Authors:** Jitao Zhang, Antonio Fiore, Seok-Hyun Yun, Hanyoup Kim, Giuliano Scarcelli

**Affiliations:** 1 Fischell Department of Bioengineering, University of Maryland, College Park, Maryland 20742, USA; 2 Harvard Medical School and Wellman Center for Photomedicine, Massachusetts General Hospital, Cambridge, MA 02139, USA; 3 Harvard-MIT Health Sciences and Technology, Cambridge, MA 02139, USA; 4 Canon U.S. Life Sciences, Inc., 9800 Medical Center Drive, Suite C-120, Rockville, MD 20850, USA

## Abstract

Brillouin spectroscopy probes the mechanical properties of material by measuring the optical frequency shift induced by photon-phonon scattering interactions. In traditional configurations, Brillouin spectrometers measure only one point of the sample at a time. This results in long acquisition times for mechanical imaging of large areas. In this work, we demonstrate a parallel detection configuration where the Brillouin shift of hundreds of points in a line can be measured simultaneously. In mm-sized samples, this novel configuration effectively shortens the acquisition time of two-dimensional Brillouin imaging from hours to tens of seconds, thus making it a powerful technology for label-free mechanical characterization of tissue and biomaterials.

Brillouin spectroscopy allows non-invasive measurement of material mechanical properties by measuring the frequency spectrum of acoustically-induced light scattering within a sample^1^. For this reason, for many years high-resolution Brillouin spectrometers based on Fabry-Perot interferometers have been widely used for material characterization and remote sensing^2^,^3^,^4^,^5^,^6^,^7^. In the past decade, the development of high-resolution spectrometers based on virtually-imaged phased array (VIPA) has greatly increased the spectral detection efficiency thus enabling mechanical characterization of biological tissue and biomaterials^8^,^9^,^10^. Further improvements in spectrometer performances^11^,^12^,^13^,^14^,^15^ have enabled *in vivo* measurements at safe power levels^16^,^17^ and 2D/3D imaging of biological cells^18^,^19^. However, despite the speed improvement enabled by VIPA spectrometers, Brillouin microscopy remains a slow technique compared to other imaging modalities. The optical signal of spontaneous Brillouin scattering is weak: about 100 ms are required with a few mW of illumination power focused on the sample to get sufficient signal-to-noise ratio (SNR) for accurate spectral measurements. Moreover, traditional Brillouin spectrometers can only measure one point of the sample at a time (Fig. 1a), therefore obtaining a 2D or 3D image can require a long time^18^,^19^,^20^,^21^,^22^. For many applications requiring imaging of large areas or volumes, reducing the time of data acquisition is critical. One avenue explored in recent years to address this issue has been to take advantage of nonlinear/stimulated Brillouin interactions rather than spontaneous scattering, as they potentially provide much higher energy conversion efficiency. Several techniques based on stimulated scattering phenomena have been recently demonstrated^23^,^24^,^25^,^26^,^27^,^28^; however, to date spontaneous-based protocols have remained faster than stimulated Brillouin imaging protocols in practice^29^,^30^. Considering also the added complexity and higher cost of stimulated-based setups, it is not clear whether in biological and clinical settings they will provide a net advantage.

In this work, we devised a novel configuration based on spontaneous Brillouin scattering that allows hundreds of points in a sample to be measured simultaneously using line-scanning parallel detection of scattering spectra. The schematic of this novel configuration is shown in Fig. 1b. Instead of collecting the backward scattering light as in Fig. 1a, the Brillouin scattering signal is collected with a 90-degree scattering geometry^31^,^32^. As input light, a line beam is created into the sample by focusing the incoming laser with a low-NA objective lens. This arrangement allows us to analyze multiple points along the beam line simultaneously by using a multiplexed VIPA-based spectrometer, described in Fig. 1c. With this setup, we reached the equivalent acquisition time of less than 1 ms per sample point, two orders of magnitude shorter than any previous protocols of Brillouin microscopy. A high-resolution two-dimensional Brillouin image can therefore be obtained in a matter of seconds by scanning the sample only in one dimension compared to ~hour required in traditional confocal configurations.

## Results

### Characterization of line-scanning Brillouin spectroscopy

**Figure 1:**
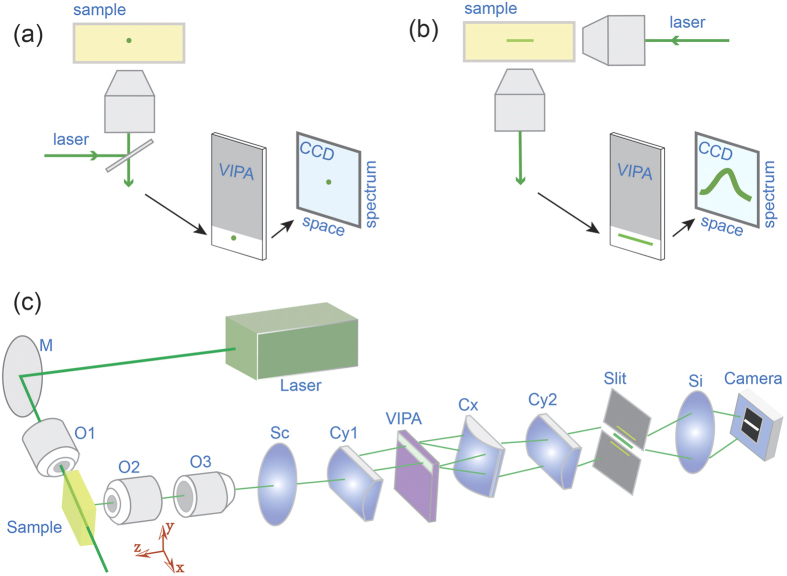
Comparison of point-scan vs line-scanning configuration of Brillouin microscopy. (**a**) The traditional configuration couples a confocal microscope with a spectrometer. A spatial point of the sample is selected by the microscope and its spectrum is measured by the spectrometer. Thus, the images are formed by scanning the sample point-by-point. (**b**) In the new configuration, by arranging the illumination path and detection path at an angle (90 degree), many points along the line beam can be measured simultaneously and the images can be formed by scanning the line beam. (**c**) Schematic of the line-scanning Brillouin spectroscopy. M: mirror; O1,O2,O3: objective lens. Sc: spherical lens for beam collimation; Cy1, Cy2, Cx: cylindrical lenses; Si: spherical lens for imaging purpose.

**Figure 2:**
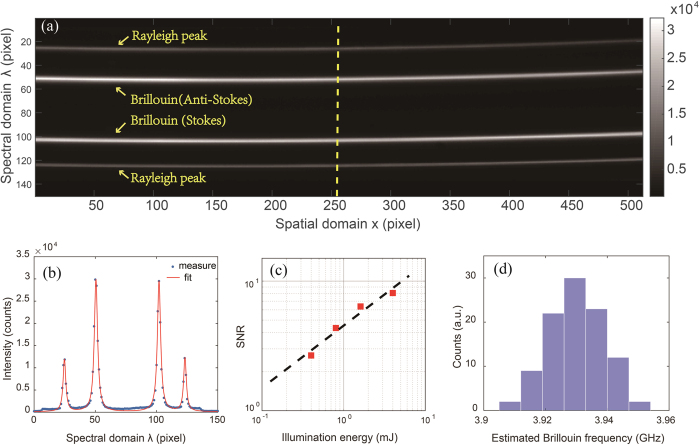
Spectral performances of line-scanning Brillouin microscopy. (**a**) Raw dispersion pattern captured by camera. The color map is expressed in terms of CCD camera counts. (**b**) Spectral pattern at a single point. (**c**) SNR vs illumination energy on a log-log plot. Square: measured data; dotted line: linearly fitted data. (**d**) Distribution of the estimated Brillouin frequencies after acquiring the same point in the sample over time.

We used a homebuilt setup of line-scanning Brillouin spectroscopy (see Methods and Fig. 1c). The spectral dispersion of the novel spectrometer was characterized first. A plastic cuvette containing pure methanol was used as sample, and the dispersion pattern captured by the camera is shown in Fig. 2a. The spectral dispersion in the figure is along the vertical axis, while the horizontal axis represents the spatial domain within the sample. The two lines at the top and bottom are due to Rayleigh elastic scattering, and thus have the same frequency as the input laser. The other two lines in the middle are Brillouin peaks of adjacent dispersion orders. Because the sample is uniform, the Brillouin Stokes and anti-Stokes signatures are two continuous horizontal lines, in the center.

Figure 2b shows the Brillouin spectrum at the location indicated by the dotted line. The spectral dispersion (red solid line) was determined by fitting the measured data with Lorentz curve. The free spectral range of the VIPA etalon was 17 GHz, and the spectral dispersion factor (i.e., ratio of frequency shift to spatial dispersion distance) was around 0.18 GHz per pixel. We measured the spectrometer finesse to be ~36 by calculating the ratio of the distance between adjacent laser peaks and their full-width-at-half-maximum. We then characterized the SNR of the spectrometer at different illumination energy (Fig. 2c). The measured data showed near square root dependence, indicating shot-noise limited behavior. The accuracy of our Brillouin spectral estimation was calculated by repeated measurements of the same point in the sample. Figure 2d shows the representative distribution of the estimated Brillouin frequency for 100 measurements at input energy of 4 mJ. It has a Gaussian distribution of standard deviation of 8.5 MHz, which corresponds to the relative uncertainty of 0.22%. The Brillouin frequency of methanol (Fig. 2a,b) as well as several other liquids (Supplementary Fig. S1) was measured and is consistent with previously published results^10^,^21^,^33^,^34^.

**Figure 3:**
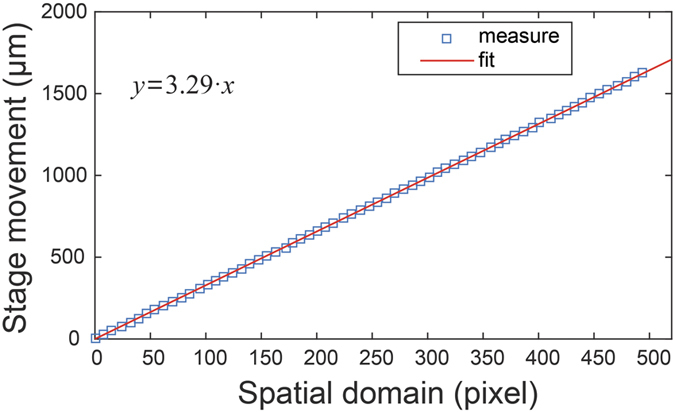
Spatial performance of the Brillouin microscopy. The step movement of the knife-edge at the sample plane (vertical axis) was monitored by the spectrometer’s camera (horizontal axis). The squares are measured data and the red line is linearly fitted data. The slope of the fitted line shows 3.29 microns per pixel.

In general, the spatial resolution of the line-scanning configuration is determined by both the illumination numerical aperture (NA) and detection NA. For our current setup, since the illumination NA (0.0175) was much smaller than detection NA (0.1), the lateral resolution (x- and y-direction) was determined by the detection NA and the axial resolution (z-direction) was determined by the illumination NA. In order to characterize the spatial performances of the setup, we placed a knife-edge right after the cuvette and in front of the detection objective lens. A translational stage carrying the knife-edge was moved in x-direction with step size of 25.4 μm, and the corresponding image of the knife-edge was monitored by the spectrometer’s camera. The result is shown in Fig. 3. The measured data was linearly fitted, and thus the spatial resolution (x-direction) was calculated as 3.29 μm per pixel close to diffraction limit. Our camera had 512 by 512 pixels in total, which corresponds to 1.68 mm field of view in the sample plane. Across the field of view, the spectral line was not perfectly straight but curved due to the deviation of the incident angles of off-axis points. This curvature may result in variation of the spectral dispersion factor at different points in the sample and reduce spectral extinction by making it difficult to block the elastic frequency using a spatial filter. To reduce the curvature, we used a high-NA tube lens in the microscope to minimize magnification before the spectrometer; then, we restored the desired image magnification with a cylindrical lens after the VIPA. In this way, we reduced the variation of the spectral dispersion factor to less than 3 MHz per pixel within more than 1-mm spatial field of view. For simplicity, we will ignore this effect and use the same spectral dispersion factor for all points in the following. For more accurate estimations, a characterization of the spectral dispersion at all points could be performed.

### Two-dimensional and three-dimensional imaging

**Figure 4:**
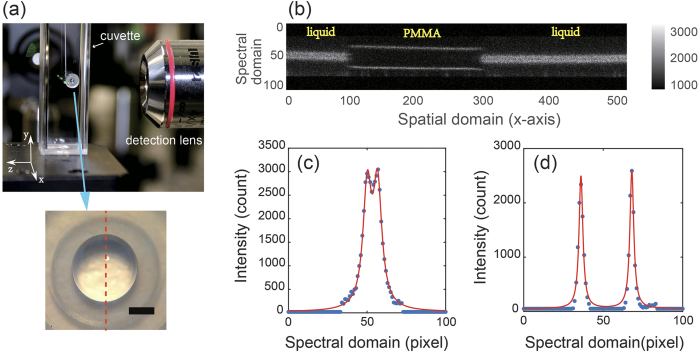
Experimental configuration and measurements. (**a**) Picture of the PMMA lens. The picture was taken before filling cuvette with index-matching liquid. The lower panel shows a zoomed-in picture of the PMMA lens. Scale bar, 500 μm. (**b**) Snapshot of representative Brillouin pattern acquired by the camera during measurement. (**c**) Brillouin spectrum of PMMA. (**d**) Brillouin spectrum of index-matching liquid. The dots and solid curves are measured data and fitted data, respectively.

We measured the Brillouin shift of an aspherical lens made of PMMA in the setup shown in Fig. 4a. The PMMA lens was placed into a plastic cuvette filled with index-matching liquid. The laser power was 70 mW, and the exposure time of the camera was 0.1 second. When the sample was immersed into the liquid, it was almost invisible by naked eye because of the refractive index matching. However, since the stiffness of the sample and the matching liquid were different, we could easily distinguish them by their Brillouin signatures. Figure 4b is a snapshot of the representative signal acquired by the camera, in which only Brillouin frequency components were shown and the elastic frequencies were blocked by a slit. The Brillouin signatures of the matching liquid surrounding the PMMA lens are apparent. At the interface of different materials, there may be cross-talk between two Brillouin signatures corresponding to each material. This cross-talk effect will introduce an ambiguous region at the interface if the two materials have similar Brillouin shift. Because the PMMA and the liquid had distinct Brillouin shifts, we were able to quantify the ambiguous region to be within 2 pixels, i.e. 6.58 μm. Figure 4c,d showed the Brillouin spectra of the index-matching liquid and PMMA at single point, respectively. Using the characterization data of the spectral dispersion, the Brillouin frequency shifts of PMMA and the matching liquid were determined to be 11.32 GHz and 9.11 GHz, respectively. The measured PMMA data is consistent with published result^35^.

**Figure 5:**
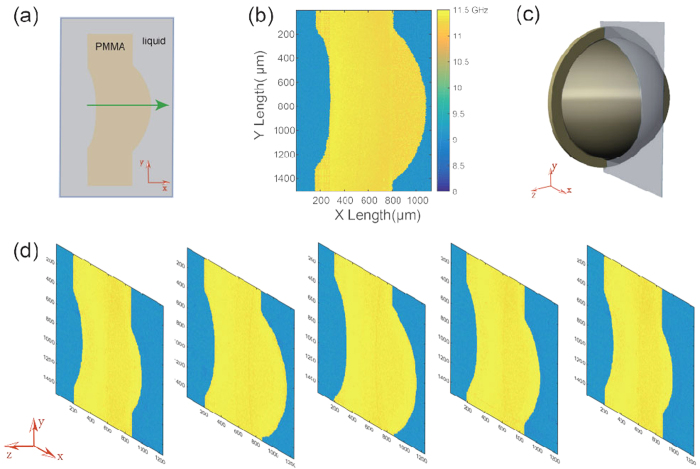
2D and 3D Brillouin images of the PMMA lens. (**a**) Schematic of the cross-section that was scanned by the beam line. (**b**) Cross-sectional Brillouin image of the PMMA lens at the location indicated by the dotted line in Fig. 4a. The colorbar represents the Brillouin frequency shift with a unit of GHz. (**c**) Schematic of the 3D scanning of the PMMA lens. 3D images were obtained by scanning the cross-section in z-axis. (**d**) 3D Brillouin imaging of the PMMA lens. Each slice represents a cross-section image of the PMMA lens at a location along z-axis.

Figure 5 shows 2D and 3D Brillouin images of the PMMA lens. The cuvette containing the lens was carried by a vertical motorized translation stage, which enabled us to scan the sample in y-direction. A LABVIEW program was developed to synchronize the stage translation and camera acquisition so that the scanning could be carried out automatically. The speed of the stage was set as 50 μm/s and 300 frames were captured. Thus, within 30 seconds we acquired ~100,000 points of the sample within a field of view of 1.1 mm by 1.5 mm, a task that would require more than 1 hour in previous confocal Brillouin microscopes. Figure 5a indicates the scanned cross-section along the dotted line of Fig. 4a. The green line indicates the illumination beam. Figure 5b shows the 2D imaging of the PMMA lens immerged in the matching liquid based on the measured Brillouin frequency shift. The interface between the PMMA lens and the matching liquid could be clearly seen from the image, and the inner region of the PMMA lens was pretty uniform. We then demonstrated the capability of this spectrometer to do a rapid 3D imaging by use of the scanning method shown in Fig. 5c. Figure 5d shows five slices of obtained cross-section images as we moved the PMMA lens along z-axis using another translational stage.

## Discussion

To understand advantages and limitations of this new configuration, we started by comparing the spectral efficiency of angled geometries with traditional setups using confocal epi-detection configuration. If the illumination beam is vertically polarized and thus perpendicular to the scattering plane, the differential cross-section is not dependent on the scattering angle^36^, thus configurations with angled illumination-detection paths have the same differential cross-section as the epi-detection. However, the angled geometry will result in diminished geometrical efficiency per single point (i.e. not counting the advantage due to the massive parallelization of the measurement). The collected scattering power can be written as *P* = *I*_*ill*_ · *V* · Ω · *R*, where *I*_*ill*_ is the intensity of the illumination light, *V* is the interaction volume of the scattering, Ω is the collected solid angle, and *R* is the scattering coefficient, which has a unit of *m*^−1^ and can be considered as constant here. In orthogonal configuration, the interaction volume can be approximated by a cylinder with radius *r* = 0.61*λ*/*NA*_*col*_ and length *l* = 0.61*λ*/*NA*_*ill*_, where *NA*_*ill*_ and *NA*_*col*_are the NA of the illuminating objective lens and collecting objective lens, respectively. The collected solid angle depends on the collecting numerical aperture 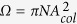. Therefore, the collected scattering power is 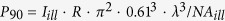. For epi-configuration, instead, the interaction volume is approximately 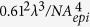, where *NA*_*epi*_ is the NA of the objective lens, and the collected solid angle is 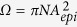. Thus, the collected scattering power is 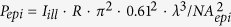. With same illumination intensity, the ratio of the collected power between two configurations turns out to be 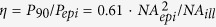. In orthogonal configurations, low *NA*_*ill*_ is usually preferred in order to generate a long illumination beam line uniformly across the field of view. For example, we used *NA*_*ill*_ = 0.0175 that corresponds to a 1.74 mm usable Rayleigh range. Comparing our particular experimental configuration with epi-configuration with *NA*_*epi*_ = 0.1 (i.e. providing the same resolution in x-direction), the ratio is 34.8%. Our calculation is consistent with the prediction by other work^37^ where a different combination of objective lenses was used. Importantly, this calculation is consistent with the experimental results in Fig. 2 and with other epi-detection results^18^. The scattering efficiency in orthogonal and epi-detection is determined by the overlap between the illumination volume and detection volume, therefore one can design angled-geometries to be as efficient as epi-detection per single point measurement to maximize the parallelization advantage. Including the line parallel detection, the new method can accomplish the scan of a mm-sized sample with few-micron resolution within tens of seconds compared to >1 hour in epi-detection. Our method shares the same principles of light-sheet microscopy^38^ and 2D Raman spectroscopy^39^; In the orthogonal configuration of our current setup only samples accessible from both sides and with thickness of ~1 mm size (due to the limited Rayleigh range of the illumination beam) are measurable. This limitation can be solved with dual-beam illumination, which has been developed for light-sheet microscopy^40^.

In terms of background rejection, the angled configuration has inherently less background noise than epi-detection. In standard confocal configuration, back reflections of the illuminating light are easily coupled into the spectrometer and contribute to the background noise^11^. In the line-scanning configuration, the illumination and detection path are arranged orthogonally, thus the back reflection can be completely avoided. Furthermore, since only the region that is illuminated by the line beam produces Brillouin scattering signal, the line-scanning configuration enables optical sectioning as in the confocal configuration. On the other hand, in order to have parallel detection only one VIPA stage can be used; this limits the extinction ratio of the spectrometer to ~30 dB. This makes it challenging to measure interfaces or optically turbid samples, such as biological tissue. To improve the overall extinction ratio of the instrument, the line-scanning configuration can be combined with existing methods to suppress the background noise such as apodization^18^, narrow band-pass filtering^41^,^42^, and gas-chamber narrow absorption filtering^13^.

## Methods

### Brillouin scattering

Spontaneous Brillouin scattering is the scattering of light from sound waves (acoustic phonons). The elastic modulus *E* of an isotropic material is linked to the Brillouin frequency shift *v*_*B*_ by the formula


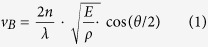


where *n* is the refractive index of the ambient, *λ* is the wavelength of the light source, *ρ* is the density of the material, and *θ* is the scattering angle. By measuring the Brillouin frequency shift via a spectrometer, the mechanical properties of the sample can be determined.

### Experimental setup

The light source was a single mode 532-nm cw laser (Torus, LaserQuantum). The beam from the laser head was focused by the objective lens O1 (NA = 0.0175) to generate a line beam and illuminated the sample. At 90 degree, the scattering light along the beamline was first imaged by a pair of objective lens O2 and O3 (both were 4X/0.1NA), and then collimated by a spherical lens Sc (f = 400 mm). The collimated light was then focused onto the entrance of a VIPA (FSR = 17 GHz, finesse = 36) by a cylindrical lens Cy1 (f = 200 mm). The dispersed light after the VIPA was imaged onto the plane of a slit by a pair of cylindrical lens Cx (f = 1000 mm) and Cy2 (f = 400 mm). This plane was re-imaged onto the camera (iXon, Andor) by a spherical lens Si (f = 60 mm). The slit works as a spatial filter in the measurement. By adjusting the aperture of the slit, we could block the undesired frequency and only let Brillouin frequency components pass (Fig. 1c).

### Calibration

The calibration of the setup consists of two parts: spectral calibration and spatial calibration. The spectral calibration can be performed at each pixel (corresponding to one point of the sample) of the spectral line using measured spectral patterns of reference materials with known Brillouin shift (e.g., Supplementary Fig. S1). If Rayleigh peaks are cut by spatial filters, two unknown parameters need to be estimated: FSR and spectral dispersion factor. Therefore, the measurements of two samples of known Brillouin shifts are sufficient for calibration purposes. More reference materials will be needed if the spectral dispersion is not constant across the different spectral orders. The calibrated results can be further confirmed by comparing with the distance of two adjacent Rayleigh peaks by opening the slit. The calibrated FSR and spectral dispersion factor are then used to determine the unknown Brillouin shift of the samples under examination. The spatial calibration was carried out by translating a knife-edge with micrometric accuracy along the sample plane; the results are shown in Fig. 3, which indicates a good linear relationship.

### Brillouin spectrum linewidth

The overall linewidth of the measured spectra is mainly determined by three contributions: intrinsic linewidth of the sample, limited finesse of the VIPA spectrometer, and spectral broadening from optical componenets (e.g. limited NA of the optics and other distortion effects). In our experiment, the limited finesse of the spectrometer is the largest broadening factor. For example, the intrinsic linewidth of methanol is about 0. 33 GHz^33^, and the linewidth of the spectrometer is about 0.47 GHz considering the FSR of 17 GHz and experimentally-measured finesse of ~36. Therefore, the minimum linewidth of the measurement is the convolution of intrinsic linewidth and finesse-limited linewidth, i.e., 0.557 GHz. In experiment, we measured 0.566 GHz at the center of the spectral line, very close to the expected limit. At the tail of the full field of view, small broadening effects due to optics elements are observed and the linewidth is ~3.5% broader than that at the center of the field of view.

### Sample preparation

The PMMA lens was produced for cell phone’s camera and provided by an OEM supplier. The plastic cuvette had a size of 10 mm by 10 mm. To make the PMMA lens suspend in the central region of the cuvette, we first attached the margin of the PMMA lens to the tip of a syringe’s needle using optical adhesive, and then fixed the end of the needle to the wall of the cuvette. The liquid (Cargille Lab) had the refractive index of 1.4917 in order to match the PMMA. The motorized translational stage (T-LSM025A, Zaber) had a 25-mm traveling range and RS-232 control. The maximum speed can be as fast as 7 mm/s.

## Additional Information

**How to cite this article**: Zhang, J. *et al.* Line-scanning Brillouin microscopy for rapid non-invasive mechanical imaging. *Sci. Rep.* **6**, 35398; doi: 10.1038/srep35398 (2016).

## Supplementary Material

Supplementary Information
