# Supplementary material for: Line-scanning Brillouin microscopy for rapid non-invasive mechanical imaging
Source: Sci Rep. 2016 Oct 14;6:35398. doi: 10.1038/srep35398 (PMC5064313; doi:10.1038/srep35398)
Supplement: Supplementary Information [file srep35398-s1.pdf]

## Supplementary Information

# Line-scanning Brillouin microscopy for rapid non-invasive mechanical imaging

**Jitao Zhang[1], Antonio Fiore[1], Seok-Hyun Yun[2,3], Hanyoup Kim[4], and Giuliano Scarcelli[1,*]**

[1]Fischell Department of Bioengineering, University of Maryland, College Park, Maryland 20742, USA

[2]Harvard Medical School and Wellman Center for Photomedicine, Massachusetts General Hospital, Cambridge, MA 02139, USA

[3]Harvard-MIT Health Sciences and Technology, Cambridge, MA 02139, USA

[4]Canon U.S. Life Sciences, Inc., 9800 Medical Center Drive, Suite C-120, Rockville, MD 20850, USA

[*]Corresponding: scarc@umd.edu

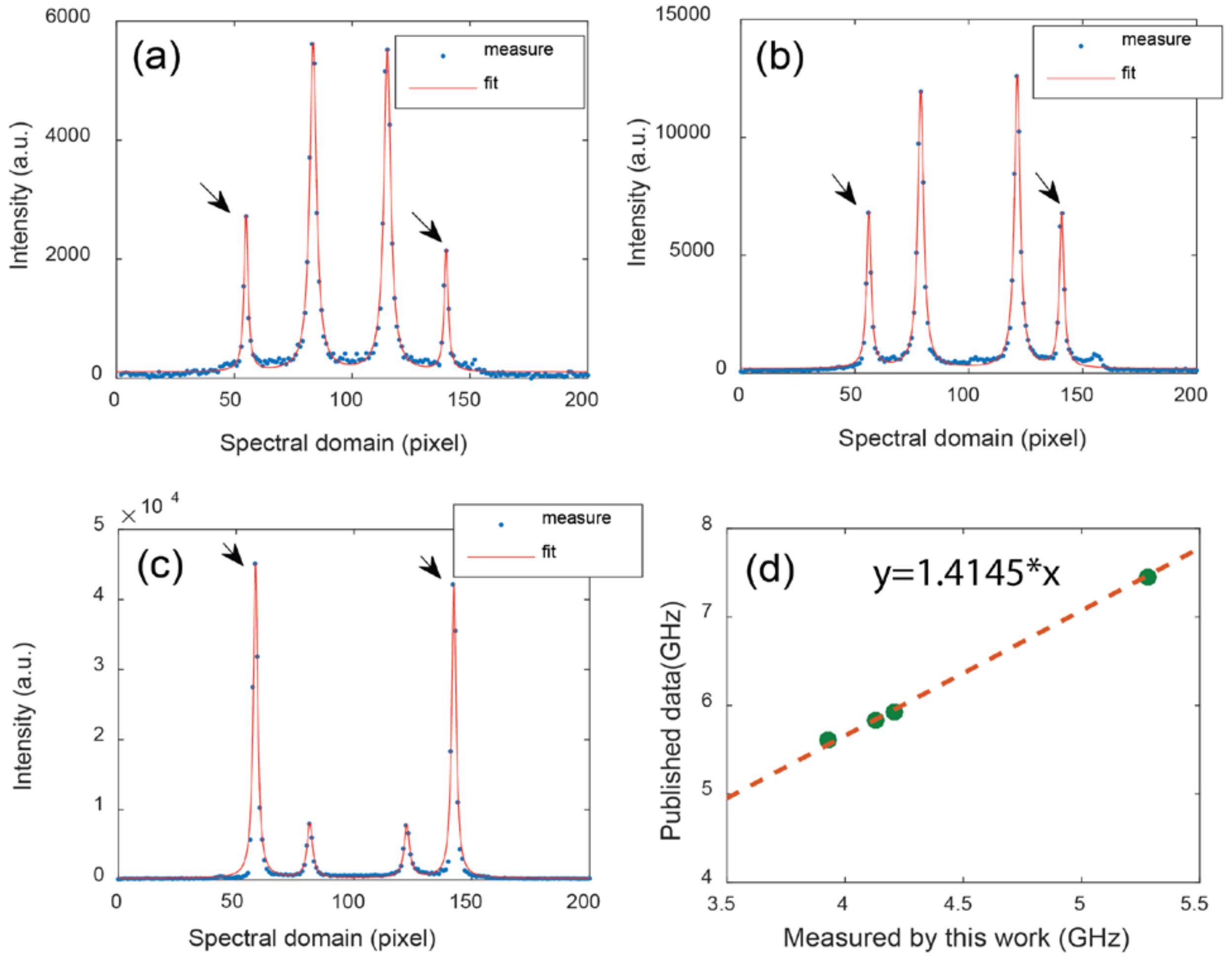


**Supplementary Figure S1**. Measured Brillouin frequency shifts of (a) deionized water, (b) ethanol, and (c) acetone by line-scanning Brillouin microscopy are 5.28 GHz, 4.13 GHz, and 4.21 GHz, respectively. The arrows indicate the Rayleigh peaks; the peaks in between Rayleigh signals are Brillouin peaks. We compared these results (plus methanol from main text) with published data[1-4] measured at 180-degree geometry, and showed an excellent correlation between published data and our measurements (R =0.99). The linear relationship with slope of $\sqrt{2}$ is due to the expected difference of scattering geometries (i.e., 180-degree vs 90-degree).

## Reference


1. Faris, G. W., Jusinski, L. E. & Hickman, A. P. High-resolution stimulated Brillouin gain spectroscopy in glasses and crystals. *J. Opt. Soc. Am. B* **10**, 587–599 (1993).
2. Goodwin, R. D. Methanol Thermodynamic Properties from 176 to 673 K at Pressures to 700 Bar. *J. Phys. Chem. Ref. Data* **16**, 799–892 (1987).
3. Iglesias, M. et al. Thermodynamic Properties of the Ternary Mixture Acetone +Methanol + Ethanol at 298.15 K. *J. Chem. Eng. Data*. **43**, 776-780 (1998).
4. Koski, K. & Yarger, J. Brillouin imaging. *Appl. Phys. Lett*. **87**, 061903 (2005).
